# Mental Labour; Its Effects on the Blood
*Read before the Medical Society of London, November 29th, 1856.


**Published:** 1857-01-01

**Authors:** Theophilus Thompson

**Affiliations:** Physician to the Hospital for Consumption and Diseases of the Chest, &c.


### Art. VI.—MENTAL LABOUR; ITS EFFECTS ON THE BLOOD.*

BY THEOPHILUS THOMPSON, M.D., F.R.S.,

Physician to the Hospital for Consumption and Diseases of the Chest, &c.

The progress of civilization, notwithstanding the incalculable benefits with which it is attended, nevertheless involves many countervailing evils; but it is impossible to direct much thoughtful observation to the present condition of the world, without being impressed with the conviction that, amongst the causes of disappointment associated with modern advancement, there are some which are not necessarily irremediable. Among the subjects for inquiry intimately related to this topic, there are few which are more likely to reward investigation than those which regard the reciprocal influence of mental and physical conditions.

The invention of printing, by the aid of which civilization has been pre-eminently promoted, in giving permanence to acquired knowledge, has so raised the general standard of attainment and taste, that distinction in the walks of literature or science can rarely be attained, excepting by an amount of assiduous labour such as can be endured only by individuals whose constitutions are distinguished by a peculiar combination of physical and intellectual energy. As the number of aspirants to distinction multiplies, sympathy becomes more intense, taste more fastidious, competition more keen, and the craving for intellectual enjoyment increasing with the means for its gratification, an honourable association with the highly-cultivated classes of society cannot be maintained without considerable mental effort. The struggle for eminence in any department of intellectual labour involves a concentration of mind on the special subject, which, if long protracted, is particularly calculated to induce disorder.

The tendency to such concentration, increased by habit, becoming in some individuals almost irresistible, proves seriously detrimental to the mental and bodily health.

Whether the continuous exertion of one faculty acts unfavourably by withdrawing the circulating fluid from other organs, and so disturbing the healthy balance, or whether excessive action of a particular faculty exhausts some special material of the blood, is a question which cannot, perhaps, in the present state of our knowledge, be positively determined; but instances of morbid condition thus induced are continually presenting themselves to the physician.

* Read before the Medical Society of London, November 29th, 1856.

Intellectual, like muscular action, probably involves an expenditure of living material, and introduces a changing series of particles—those which have been used giving place to others, which come with the energy of new life to perpetuate the action. There may be decay from stagnation—there may be waste from persistency, undue haste or intensity, especially in creative efforts. It is only when the function is performed in a calm and equable manner that the equilibrium of expenditure and supply is maintained, and that power is preserved and increased.

Not long since an account-keeper from a large public establishment, where he had been accustomed to work without intermission twelve hours daily, came to my consulting-room almost in tears, saying he was fit for nothing, feeling as though cut off from everything, and as if, when he attempted to fix his attention on any subject, some indescribable influence drew it away—a distressing sensation in the chest, and tingling of skin, as though the bed was full of fleas, often keeping him awake; and his sleep disturbed by frightful dreams. His height was 5 feet 5 inches; weight 129 lbs.; appetite good; tongue natural; bowels regular; urine slightly acid, of good colour, its specific gravity 1025, containing a very few small oxalate of lime crystals, and scanty lithates. Pulse 88, not strong; skin moist. There was a strong continuous venous hum in both jugular veins, heard the more distinctly on checking respiration, but not overpowering the sound of the arterial pulse. When he cuts himself in shaving, the blood flows freely, and is with difficulty stanched. Under the microscope, a remarkably small number of pale-coloured corpuscles in proportion to the red discs, was observable—a peculiarity which has repeatedly arrested my attention in cases associated with venous murmur, remarkably contrasting in this respect with the condition of blood ordinarily present in pulmonary consumption. Assuming that the relative proportion of pale corpuscles to the red is in phthisis one in ten, in healthy individuals one in fifty, the blood of patients belonging to the class under consideration has appeared to me to exhibit a proportion of about one in two hundred. I am inclined to suspect that this peculiarity is associated with deficient quantity of fibrin—a condition the reverse of that which usually obtains amongst consumptive individuals. But observations require to be extensively multiplied before we can safely venture in this matter to propound a rule.

Some of the evils briefly noticed in my preliminary remarks press with peculiar force on the clergy. Often beginning life with anxious competition for university honours, they pass at an early period of their career into responsible duty, debarred by conventional rules from many innocent recreations, subjected to unusual restraint of demeanour, restricted to intellectual pursuits which overtax particular faculties, to the comparative neglect of others, and exposed in more than the average degree to the wear of sympathy, it is not surprising that the younger clergy, before their constitutions are consolidated, become so often the subjects of bodily infirmity, their nervous system unduly susceptible, and their minds too easily accessible to the delusions of pseudo-science and quackery.

A popular clergyman, of active sensitive mind, aged forty-three, but from his grey hair and general aspect likely to be considered fifty, had been, since he left the university, affected in a great degree with sleeplessness; often for weeks together not sleeping at night more than two hours. He could not refer this inaptitude for sleep to any physical cause. The alvine and renal evacuations were natural, and muscular strength good; but the pulse was rather weak; there was a marked murmur in the right jugular vein, and he complained of deficient intellectual power as respected the suggestion of ideas. I prescribed cod liver oil, and nitro-hydrochloric acid. The pulse improved, and in three weeks his average sleep was five hours a night. I then administered phosphate of iron, with phosphoric acid. In two or three months he recovered a fair degree of health, the venous murmur was scarcely audible, and the sleepless nights and feeling of mental sterility occurred only occasionally after extra efforts of composition.

In the summer of 1855, after anxiety connected with schools, and claims on his sympathy in consequence of affliction among friends, a relapse occurred. I sent him to the country with injunctions to avoid as much as possible the society of civilized man. He at first gained little ground, being much engaged in discussion with intelligent acquaintances; but on removing to a more secluded spot to vegetate in the open air, his powers of sleep and composition returned.

The nature of ordinary cases of the kind referred to, may be illustrated by reference to those of an extreme character: a sudden shock to the nervous system, whether physical or mental, tending to induce conditions more severe in degree but analogous in nature to those resulting from the slighter, but more continuous series of shocks produced by the wear of anxious intellectual effort and disturbed sympathy.

A few years since, an express train on its way to meet the Queen, ran into another, and many of the passengers were injured. A lady, aged 33, had her head severely wounded, the scalp laid bare, one ear nearly cut off, the teeth knocked in; Mr. Bransby Cooper, who attended her, said she must have had an unparalleled constitution not to have sunk. There was no great loss of blood, but she suffered for some time after the accident from hysterical cough, and inability, as she said, to swallow except by sips—became thin and pallid—the heart excessively irritable. These conditions, accompanied with a venous murmur, continued last year, and although materially benefited by the administration of zinc and cod-liver oil, they still continue in a considerable degree. The symptoms in this patient are not fairly referable to loss of blood. Her sister, who suffered from the same catastrophe, lost much more blood; but her subsequent ailments, although presenting slight analogies, were much less severe, and not accompanied with venous murmur.

I would here present a case illustrative of the effects of brain-shock on the blood, which occurred in the practice of Sir Henry Marsh:*

"A young and beautiful woman in the middle rank of life, highly but self-educated, of great mental endowment, of admirable taste, and strong sensibility and attachment, was unconsciously the one by whose hand a poisonous dose was administered to her sole surviving parent, to whom she was attached with all the fervour and devotedness of a daughter's love. The phial contained an ounce and a half of laudanum; it was given by mistake for a senna draught. When presented to him by his daughter, he tasted it, and said he did not like it, and would not take it. He had not been in good health; it was with much entreaty he was ever prevailed on to take the medicines prescribed. She urged him in terms the most affectionate and persuasive to take his draught; he replied, 'Dearest, you know I never can refuse you anything,' and swallowed it. Three hours passed away before she was aware of her terrible mistake. She was aroused to it by the state of stupor into which her father had fallen, when it flashed across her mind. She found the senna draught which she had intended to have given untouched; she also found the word 'poison' printed in large letters on the empty phial. The shock to her mind was terrific. She became like one insane. All possible means were employed to save the life of the poisoned man, but they were employed too late. He died profoundly comatose at the end of a few hours. From the moment of his last breath a change came over her. She was lost to all knowledge or notice of persons and occurrences around; she lay like a statue, pale and motionless. Food she never took, excepting when it was placed upon her tongue. The only sound which escaped her lips was a faint yes or no. When asked what ailed her, she would place her hand upon her heart. Her extremities were cold. She

* Dublin Quarterly Journal of Medicine, August 1st, 1853, p. 1.

sighed and shivered frequently, and dosed brokenly and protractedly. To her, the world, and all things in it, were a blank. Tonics and stimulants were administered, air and scene were changed, kind and compassionate relatives and friends tried and tried in vain to rouse and console; she pined away, and nought but a breathing skeleton remained. She lingered on with very little variety or alteration of symptoms for ten months. Before her dissolution she became œdematous. The swelling, soft and transparent, was first perceived in the lower extremities, but gradually progressed upwards. It became apparent on the backs of the hands, along the arms, and ultimately it was universal. All the viscera, spinal, cerebral, thoracic, and abdominal, were patiently and minutely examined. No trace of organic change of structure could be detected. There was a copious effusion of thin transparent serum into every cavity—into every serous tissue. The pericardium was separated from the heart by an abundant effusion. The large amount of the dropsical effusion contrasted strangely with the extreme attenuation. In this case, to repress the increasing dropsy, ovipuncture had been several times practised, always with relieving effect; even with this deduction, the viscera appeared as it were bathed in water. This poor patient, beaten down in mind and body, breathed her last without a moan or a painful struggle. The mental shock had paralysed the vital actions, an evidence that, in real life, events do occur which transcend even the highest flights of fiction. An almost total suspension of nutrition, sanguification, and vascular energy characterized this case. The result was universal dropsy consisting in the thinnest serosity."

I have known an engraver, after working long and successfully on cathedral drawings, unable to sleep soundly on account of being haunted with architectural lines; but he went out for a walk among the mountains, recovered his capability of sleeping, and came back home "hard as nails."

Another engraver, (whom I attended,) anxiously and continuously engaged in the same department of work, in which he had greatly distinguished himself, became unable to recognise his own house.

A similar impairment of memory, accompanied as in the preceding instances with venous murmur, I have observed in some benevolent and conscientious individuals inordinately engaged in carrying out some scheme of philanthropy;—in others associated with mental depression, taking the direction of some unsound or narrow religious dogma;—and in professional men, worn with the stir and anxiety of life. In all such instances, measures calculated to enrich the blood have proved an important auxiliary to those which have respected mere change of scene and occupation.

The instances specially present to my mind in making this communication are not remarkable for any impairment of digestive function, and the attendant impoverishment of blood seems to be a result of nervous exhaustion. The somewhat pallid cheeks, the languid eye, the venous murmur, are in harmony with the intellectual manifestations,—in some sleeplessness,—in some inertness. The original writer complaining of a peculiar sterility of mind, and the close reasoner becoming fragmentary and unconnected in his trains of thought.

In all the conditions referred to there is a general analogy, but the special manifestations vary with the temperament of the individual patient. In the active and the sanguine, for example, sleeplessness is common, and a more than ordinary readiness to adopt any fashionable heresy in pseudo-science or theology. In persons of a more phlegmatic disposition there is induced great indisposition for exertion, a gloomy view of events, and perhaps a desponding estimate of their religious condition. When, as usually happens, duodenal indigestion is superadded, habitual depression of spirits is common, and oxalate of lime crystals may often be detected in the urine.

The amount of labour which different individuals can bear without such injury as we have described varies. Indeed, the mere amount is only part of the explanation—*anxiety*, *hurry*, and *exclusiveness* of work being more injurious than quantity. Work which is successful, varied, and pursued without hurry, although carried to a considerable extent, may be not only innocuous, but useful and even necessary. Indeed, without some degree of intellectual exercise, the body itself would languish. But there is a limit with every individual which cannot be safely passed; and it is the part of a wise man to watch, with a view to counteraction, the earliest indications of exhausting effort.

As respects treatment, those measures are most salutary which tend to enrich the blood. Chalybeates are sometimes useful; but in many instances they tend to increase irritability, and cannot, at least at the commencement, be safely employed. A course of cod-liver oil is seldom inappropriate, and this remedy may often be advantageously administered in combination with nitro-hydrochloric acid, especially when the appetite is defective, or the oxalic acid diathesis is present. To the subject of the importance of the remedial administration of oils, I am anxious to invite particular attention. About eighty years since cod-liver oil was largely employed at Manchester in the treatment of rheumatism, but was, after a time, superseded by other more agreeable medicines. The mode of action of the remedy was not then ascertained, and its proper place as a medical agent was not determined. During the last twelve years the value of this medicine in the treatment of consumption has been established. With the aid of the medical press, observations on its effects by different practitioners have been extended and compared, and we have found reason to conclude that its efficacy in consumption depends not on any specific adaptation to that particular disease, but on qualities which render it of equal or superior service in other disorders presenting points of resemblance as respects some peculiar conditions of the blood, which the administration of the remedy is specially calculated to correct.*

Change of occupation and scene is of paramount importance. When the condition is not one of extreme exhaustion, an energetic tour is desirable; in the more advanced cases, the disinclination for such an effort being extreme, or the excitability great, such a measure must not be abruptly commenced; perfect repose for a time may then be expedient, and habits of activity gradually adopted. When intellectual work is resumed, many precautions should be observed. The times, amount, and method should be regulated, and probably even the posture in which mental processes are carried on. Analysis being with many students best effected in the recumbent posture, and composition while walking. The importance of exercise in the open air can scarcely be over-estimated. Indeed, although instances of the class described in this communication occur not unfrequently in persons careful to observe ordinary hygienic precautions, yet there are others in whom an amount of nervous irritability, associated with the other symptoms, may be mainly attributed to a neglect of muscular exercise, the special remedy for nervous excitability.

I have for various reasons expatiated on the class of cases associated with venous murmur, and other signs of anæmia or spanæmia—1st. Because they seem to me to indicate an order of succession in the phenomena not generally recognised, pointing to the conclusion that the brain may sometimes impoverish the blood, before the condition of the other organs disturbs the brain. 2ndly. Because such instances of cerebral exhaustion are those most amenable to treatment. 3rdly. Because the probable hereditary transmissibility of such conditions makes them of incalculable importance to the community.

It is a common remark, that the type of disease at different periods varies. In tracing some past eras of disease (speaking in general terms) we may conclude that the plethoric condition, prevalent in the 17th century, gave place in the 18th to gastric congestion. This condition has now ceased to predominate, and

* *Vide* "Clinical Lectures on Pulmonary Consumption." By Theophilus Thompson, M.D., F.R.S. London. 1854. pp. 67–85.

we have perhaps entered on an anæmic era—a state peculiarly unfavourable to the manifestation of power and to the production of great men.

For all classes, but especially for the professional, a prudent regard should be had to the probable production and ready aggravation of the anæmic condition from causes associated with the laws of mind. A different training, physical and intellectual, must be adopted by our clergy, if they are to be kept in the foreground of the domain of thought, and to be suffered to direct those energies of social life which it is our part, as medical men, to cherish.

As respects the application of medical theories, a wider range of thought should be pursued. The modifications of many prevalent diseases may have reference as much to mental states as to hygienic conditions of atmosphere and diet. Every remediable infirmity is a violation of the law of progress. If medicine is to render to the community a full amount of good, it must be not simply in treating the maladies of individual patients, but in ministering to the conditions which disturb the vitality of the race, and, thus whilst improving our appliances for daily work, remembering that we are enlisted in the service of mankind, we may make posterity our debtors.

---

